# Oncogenic Tyrosine Phosphatases: Novel Therapeutic Targets for Melanoma Treatment

**DOI:** 10.3390/cancers12102799

**Published:** 2020-09-29

**Authors:** Elisa Pardella, Erica Pranzini, Angela Leo, Maria Letizia Taddei, Paolo Paoli, Giovanni Raugei

**Affiliations:** 1 Department of Experimental and Clinical Biomedical Sciences “Mario Serio” University of Florence, Viale Morgagni 50, 50134 Florence, Italy; elisa.pardella@student.unisi.it (E.P.); erica.pranzini@unifi.it (E.P.); angela.leo@student.unisi.it (A.L.); giovanni.raugei@unifi.it (G.R.); 2 Department of Experimental and Clinical Medicine, University of Florence, Viale Morgagni 50, 50134 Florence, Italy; marialetizia.taddei@unifi.it

**Keywords:** melanoma, protein tyrosine phosphatase, PTPs inhibitors, melanoma immune infiltrate

## Abstract

**Simple Summary:**

Targeting oncogenic protein tyrosine phosphatases (PTPs) with specific pharmacological approaches has been considered for a long time a hard challenge, earning these PTPs the reputation of “undruggable” enzymes. Nevertheless, PTPs have been recognized as main targets for several diseases, including cancer, and great efforts have been made to identify novel PTPs inhibitors to fight cancer progression and metastasis formation. Here, we summarize recent evidence underlining the efficacy of this strategy for melanoma treatment. In particular, we illustrate how this approach could be applied to target both cancer cells and the immune infiltrate of tumors, providing a new promising adjuvant therapy for the treatment of melanoma.

**Abstract:**

Despite a large number of therapeutic options available, malignant melanoma remains a highly fatal disease, especially in its metastatic forms. The oncogenic role of protein tyrosine phosphatases (PTPs) is becoming increasingly clear, paving the way for novel antitumor treatments based on their inhibition. In this review, we present the oncogenic PTPs contributing to melanoma progression and we provide, where available, a description of new inhibitory strategies designed against these enzymes and possibly useful in melanoma treatment. Considering the relevance of the immune infiltrate in supporting melanoma progression, we also focus on the role of PTPs in modulating immune cell activity, identifying interesting therapeutic options that may support the currently applied immunomodulating approaches. Collectively, this information highlights the value of going further in the development of new strategies targeting oncogenic PTPs to improve the efficacy of melanoma treatment.

## 1. Introduction

Reversible tyrosine phosphorylation is one of the most important post-translational modifications, which regulates key aspects of cellular biology, such as protein stability, protein–protein interactions, and enzyme activity [1], thereby modulating the functionality of fundamental elements involved in signaling transduction of mammalian cells [2]. The intracellular tyrosine phosphorylation level is maintained by a strict balance between the activities of protein tyrosine kinases (PTKs) and protein tyrosine phosphatases (PTPs), which catalyze respectively the addition or the removal of phosphate from tyrosyl residues of their substrates [3]. An imbalance in this regulation is a well-recognized feature of several diseases, including cancer, since it induces alterations in cell growth and survival, cell migration, and tissue differentiation [4,5]. While the role of PTKs as oncogenic proteins has been largely described, allowing the development of a wide range of inhibitors already accepted in clinical use [6,7], an effective strategy to modulate PTPs for cancer therapy has yet to be identified [8].

PTPs are a large family of proteins consisting of 107 members that can be classified into four families (class I, II, III, and IV) according to the amino acid sequence at the catalytic domains [9]. Originally, PTPs were described to exclusively have a role as tumor suppressors, counteracting the activity of PTKs. However, further studies brought to light that some PTPs can also function as oncogenes depending on the availability of their functional partners and tumor type [5]. Such flexibility in functions can be explained considering that, differently from PTKs, PTPs can act both as negative and positive regulators of signal transduction pathways and can either activate or inhibit the oncogenic role of PTKs. Indeed, PTPs can exert their function by directly dephosphorylating PTKs, or, indirectly, by interfering with their downstream targets [10].

Starting from these bases, a deeper understanding of the dual role of PTPs in affecting tumor progression could lead to the development of new therapeutic strategies aimed at targeting different classes of tumors [11].

Despite the increasing evidence demonstrating the contribution of oncogenic PTPs in supporting tumor progression, the number of inhibitors available to date is still extremely limited. Primarily, this is due to the fact that PTPs have been considered for a long time as “undruggable targets”, delaying the design of pharmacological inhibitors [12,13,14]. In particular, the nature of PTP active sites represents a big challenge for the development of specific inhibitors active inside the cells [14,15]. First of all, in order to target the highly positively charged PTP active site, it would be necessary to develop negatively charged molecules, a characteristic that unfortunately strongly limits their cell permeability and bioavailability. However, promising prospects have come from the recent discovery of nonhydrolyzable, polar, and cell-permeable pTyr mimetics that gave a new chance to overcome these problems [12]. Moreover, designing specific PTP inhibitors is further complicated by the excellent conservation of the amino acid sequence inside the active site among PTPs [13]. Remarkably, although crystal structures of different members of the class-I PTPs revealed a common Cα-backbone signature, the surfaces around the PTPs’ catalytic site are characterized by distinct properties, including different topology, electrostatic potential, and lipophilic features, that can be addressed when designing novel selective inhibitors [16,17,18,19]. Furthermore, the identification of allosteric inhibitors is crucial to avoid targeting of the charged and conserved PTP active site, allowing the development of cell-permeable and selective compounds [20,21,22]. Another central obstacle is the presence of a shallow pocket at the PTPs’ catalytic site. This issue could be solved by proposing bidentate inhibitors, targeting both the active site and proximal non-conserved binding sites that are present in most of the PTPs [23,24].

Finally, another major challenge that has emerged during the screening of PTP inhibitors is the susceptibility of these enzymes to radical oxygen species (ROS), a phenomenon that in the past has often led to erroneous conclusions during investigations about possible inhibitors. Indeed, it is known that several anticancer drugs increase ROS production through redox cycling or the inhibition of ROS scavenger enzymes [25]. In analogy to other cysteine-based enzymes, the activity of PTPs is highly susceptible to oxidation of catalytic cysteine residues, which leads to the complete inactivation of these enzymes [26]. This evidence highlighted that, in order to avoid an erroneous interpretation of the mechanism of action of PTP inhibitors, their ability to generate ROS should always be evaluated both in vitro and in vivo. As far as this topic is concerned, the studies conducted in the past to analyze the mechanism of action of different types of cysteine protease inhibitors could be kept in mind as pivotal examples [27,28,29].

Noteworthy, some of the PTPs recognized to have oncogenic properties have been found to be overexpressed in highly metastatic melanoma [30,31,32,33,34,35], providing the opportunity to develop new strategies to fight this disease.

Malignant melanoma is an aggressive form of cutaneous neoplasia that derives from a series of alterations occurring in melanocytes, the melanin-producing cells resident in the basal layer of the epidermidis [36]. Melanoma retains the highest mortality rate among skin cancers and the highest potential of dissemination [37]. The majority of melanoma patients develop the cutaneous form of the disease, while non-cutaneous melanomas (which include tumors of the ocular and mucosal sites, such as anorectal, vaginal, nasal, and gastrointestinal tract) are relatively rare [38]. Both classes of melanoma are considered as a multi-factorial disease, whose pathogenesis is affected by environmental and genetic factors. However, the differential incidence of genetic alterations among melanoma subtypes and the unequal exposure to UV radiation, according to the anatomic site, strongly influence the molecular pathways involved in tumorigenesis, ultimately leading to the need for specific therapies against the different subtypes [39].

Data published in the Cancer Genome Atlas (TCGA) Network classify cutaneous melanomas into four genetic subgroups on the basis of the most frequently mutated genes involved in the mitogen-activated protein kinase (MAPK) pathway: BRAF, RAS (N-H-K), NF1, and triple wild-type (WT) melanomas [40,41]. Mutations in BRAF and NRAS are most commonly detected in primary cutaneous melanomas [42]. In particular, BRAF is mutated in about 50% of cutaneous melanomas, and among these, in 80–90% of the cases, the missense activating mutation V600E is present. Besides, NRAS mutations occur in about 20–25% of melanomas [43,44]. Following BRAF and NRAS, NF1 is the third gene most commonly mutated in cutaneous melanoma (in about 17% of the cases) and frequently co-occurs with mutations in the RASA2 gene [41,45,46]. NF1 mostly displays point mutations, which determine a loss of function with consequent constitutive activation of the MAPK and phosphoinositide 3-kinase (PI3K) pathways [47]. Interestingly, all these mutations finally result in a constitutive activation of MAPK/ERK signaling, which indeed is present in 98% of melanomas, promoting cellular proliferation, survival, and angiogenesis [48,49]. Finally, the loss of function of PTEN is observed in about 10–35% of cutaneous melanomas, where it confers resistance to BRAF inhibitors. This mutation results in a constitutive activation of the PI3K/AKT pathways, which, in turn, leads to cell growth and proliferation and to the inhibition of apoptosis [50].

Conversely, non-cutaneous melanomas have significantly lower numbers of mutations: Acral melanomas have, in about 15–20% of the cases, mutations in BRAF, NRAS, and KIT [51]; mucosal melanomas display KIT mutations in about 15% of the cases (primarily in genitourinary or anal forms) but rarely present mutations in BRAF and NRAS [52]; and uveal melanomas have distinct genomic patterns, presenting mutations either in GNAQ or GNA11 in > 90% of the cases while BAP1, SF3B1, and EIFAX are distinct subsets [53,54,55].

The involvement of protumoral PTPs in the oncogenic signaling pathways that characterize malignant melanoma may pave the way for new possible combination therapies based on pharmacological inhibition of oncogenic PTPs. Indeed, this approach could provide longer lasting therapeutic benefits through the inhibition of multiple nodes in the main oncogenic signaling pathways. In agreement with this hypothesis, Prahallad and co-authors found that the suppression of Src homology region 2 domain-containing phosphatase-2 (SHP-2) in BRAF mutant and in Vemurafenib-sensitive melanoma cells inhibits growth factor-induced drug resistance and delays the onset of spontaneous resistance [56]. Moreover, they identified the activating phosphorylation site on Tyr542 of SHP-2 as a valid biomarker to recognize patients with melanoma who have acquired Vemurafenib resistance due to receptor tyrosine kinases (RTKs) activation [56]. Moreover, due to the central role of SHP-2 in mutant KRAS-driven carcinogenesis, it has been demonstrated that the synergic inhibition of SHP-2 and MAPK/ERK kinase (MEK) results in decreased tumor growth in xenograft models of pancreatic ductal adenocarcinoma and non-small cell lung cancer, sustaining the utility of the dual SHP-2/MEK inhibition in KRAS mutant cancers [57].

The overall survival of patients diagnosed with advanced melanoma has strongly increased over recent years, thanks to the latest development of therapeutic strategies [58]. However, recurrence frequently occurs due to therapy failure leading to metastasis formation, representing the main cause of patient death [59]. For this reason, many efforts have been made to design new therapeutic approaches aimed at targeting the most aggressive stages of melanoma [60].

The data presented above underline that even if the PTP inhibitors available to date show only a mild effect on cell proliferation, future efforts could be made to use these compounds in combination with other pathway-targeted drugs to fight melanoma progression.

In this review, we will present a detailed overview of PTPs reported, up to date, to function as oncogenes in melanoma, either facilitating tumor progression or dampening the immune response. This information lays the foundation for the design of new therapeutic strategies specifically directed against oncogenic PTPs in melanoma [61].

## 2. Oncogenic Protein Tyrosine Phosphatases in Melanoma

Among the 107 known PTPs, several of them have been identified to have an oncogenic role in different types of cancers [10]. Interestingly, recent evidence highlights their importance in supporting melanoma progression, as discussed in the following sections.

### 2.1. Cell Division Cycle 25 Proteins (CDC25s)

CDC25s are a family of dual-specificity phosphatases (DSPs), known to act as key regulators of cell cycle progression and DNA damage handling, through the activation of cyclin-dependent kinase (CDK) complexes. CDC25s remove inhibitory phosphates from both threonine and tyrosine residues present on the phosphate-binding loop of CDKs [62]. Their inactivation or degradation leads to cell cycle arrest [63,64]. Hence, it is not surprising that CDC25 deregulation may lead to genomic instability and cancer transformation [64]. In humans, there are three distinct CDC25 genes (CDC25A, B, and C), which specifically dephosphorylate and activate various targets [63]. All the three isoforms are involved in tumorigenesis, even if with varying extent. Specifically, the overexpression of either CDC25A and/or CDC25B has been observed in multiple tumor types, including melanoma, where it is frequently associated with a more aggressive and metastatic phenotype [32,62,65,66,67]. By contrast, the role of CDC25C seems to be less important for tumorigenesis [68,69,70]. Interestingly, Kaplan–Meier curves of melanoma patients confirm the correlation between the higher expression of all the three isoforms and a worse clinical outcome [71,72,73,74].

Considering the importance of CDC25s in facilitating cell cycle progression and cell proliferation, these proteins are potentially very interesting targets for melanoma treatment. Determination of the crystal structures of the catalytic domain of CDC25A (PDB: 1C25) [75] and CDC25B (PDB: 1QB0) [76], alongside recent progression in bioinformatic approaches, facilitated the identification of numerous compounds with inhibitory effects on these enzymes [61].

In particular, as far as melanoma is concerned, triptolide (TPL), a diterpene triepoxide natural compound, has been demonstrated to be active in different cancers and, specifically, on the A375.S2 melanoma cell line. Treatment with this compound induces cyclin A and CDC25A inhibition, thereby causing arrest of the cell cycle in S phase. In addition, the exposure of A375.S2 melanoma cells to TPL leads to apoptosis through caspase-8, -9, and -3 activation [77]. Moreover, cantharidin (CTD), another natural compound that shares many characteristics with TPL, shows a similar ability to inhibit cyclin A and CDC25A in the A375.S2 melanoma cell line [78].

Noteworthy, Capasso and co-workers, using both experimental and bioinformatic methods, developed several quinonoid derivatives, acting as CDC25 irreversible inhibitors [63]. The mechanism of action of these compounds involves electrophilic modification or ROS-induced oxidation [79,80] of the catalytic cysteine residue in the PTP active site [81,82]. Among the molecules selected with this strategy, nine were identified with K_i_ values in the range of micro- and nano-molar and one of them (referred to as “compound 7”) showed efficacy on melanoma cells (A2058 and SAN), arresting their proliferation in G2/M phase and inducing a strong antiproliferative effect [63]. In the same paper, the authors also assessed that treatment with compound 7 stimulates the intrinsic apoptosis pathway in a caspase-dependent manner and leads to a reduction of the CDC25C protein level (and, at a lower extent, of CDC25A) [63]. However, the mechanism of action of these quinonoid-based agents may cause many different unrelated events, due to ROS reaction with other phosphatases and with unrelated enzymes. These possibilities represent a serious limit to their therapeutic applications, due to the potential toxicity.

**Figure 1:**
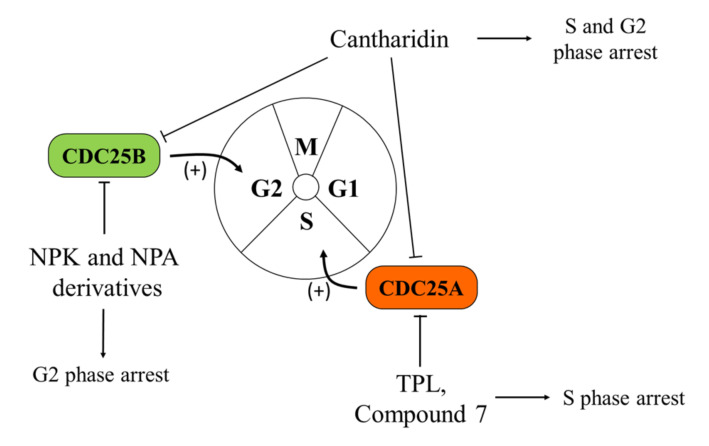
Effects of Cell Division Cycle 25 Proteins (CDC25s) targeting on melanoma cells. CDC25 phosphatases act as key regulators of the cell cycle, dephosphorylating cyclin-dependent kinases (CDK1, CDK2, CDK4, and CDK6) and cyclins (cyclin D, B, A, and E complexes). Several quinonoid derivatives, naphthylphenylketones and naphthylphenylamine derivatives, act as CDC25 inhibitors, arrest cells in the G0/G1 and G2/M phases of the cell cycle, and significantly inhibit the proliferation and colony formation ability of melanoma cells.

In order to circumvent this issue, more recently, Cerchia and co-authors performed a screening of different classes of molecules, starting from the lead inhibitor NSC28620. This approach allowed them to identify naphthylphenylketone and naphthylphenylamine derivatives, acting as CDC25 inhibitors in two aggressive human melanoma cell lines, namely A2058 and A375. In contrast with quinonoid derivatives, these compounds reversibly inhibit the enzymes (in particular, the CDC25B isoform) without generating ROS. Altogether, these characteristics make these inhibitors more interesting for possible anticancer therapy. In agreement, the reported results indicate that the treatment with these compounds affects cell cycle progression, with increased G2/M phase and reduced G0/G1 phase accumulation, by causing an increase in the phosphorylated form of cyclin-dependent kinase 1 (CDK1) [83] (Figure 1).

It should be underlined that the inhibitors so far cited are not closely specific for CDC25s and the question on their toxicity still remains open. Indeed, all the presented experiments, although showing clear cut results, are limited to in vitro melanoma models. More efforts are needed to confirm the efficacy of these new compounds as tools for melanoma treatment.

### 2.2. Low-Molecular-Weight Protein Tyrosine Phosphatase (LMW-PTP)

LMW-PTP belongs to the non-transmembrane PTPs sub-family and consists of 157 amino acids [84]. Two isoforms, generated by alternative splicing of a single gene and characterized by different activities and substrate specificities, have been found in mammalian cells [85].

Previous studies revealed that this enzyme displays a wide number of substrates, including the platelet derived growth factor (PDGF) receptor [86], insulin receptor [87], Ephrin A2 (EphA2) [88,89], and several non-receptor proteins, such as proto-oncogene tyrosine-protein kinase Src (Src) [90], focal adhesion kinase (FAK) [91], caveolin [92], signal transducer and activator of transcription 5 (STAT5) [93], β-catenin [94], and p190RhoGAP [95], thereby modulating key signaling pathways involved in tumor growth, differentiation, migration, and invasion [85,89]. In this context, it is not surprising that LMW-PTP has been found to be overexpressed in several types of human [96] and rat tumors [97], where it promotes an aggressive and malignant phenotype [98,99,100].

Recently, it has been demonstrated that LMW-PTP is overexpressed in melanoma cells, contributing to the regulation of cancer cell sensitivity toward chemo- and radiotherapy. Interestingly, it has been highlighted that the treatment of melanoma cells with morin, a non-toxic natural LMW-PTP inhibitor, is able to increase the sensitivity of tumor cells toward both dacarbazine and radiotherapy [31]. Coherently, data reported in The Human Protein Atlas database show that in melanoma patients, unfavorable prognosis is associated with high LMW-PTP expression levels, thereby confirming the role of this enzyme in regulating the in vivo survival and proliferation rate of melanoma cells [71,101]. Collectively, these findings suggest that LMW-PTP could be an interesting target to improve the effectiveness of anticancer treatment for melanoma patients that are naturally refractory to the therapies.

### 2.3. FAS-Associated Phosphatase 1 (FAP-1)

FAP-1 (or PTPN13/PTP-BAS) is a protein tyrosine phosphatase that interacts with the cytosolic portion of the Fas cell surface death receptor (FAS), whose activation leads to cell apoptosis.

FAP-1 interaction negatively regulates FAS-initiated apoptosis, preventing FAS export from the cytoplasm to the cell surface [102]. Other reported FAP-1 binding partners include the nuclear factor of kappa light polypeptide gene enhancer in B-cells inhibitor, α (IκBα), the Rho GTPase activated protein 1 (RhoGAP1), Ephrin B1, and the transient receptor potential cation channel M2 (TRPM2). In particular, IκBα is a putative FAP-1 substrate, being the only FAP-1-binding protein that is also dephosphorylated by this phosphatase [103].

Since FAP-1 negatively regulates FAS-initiated cell apoptosis, it has been suggested to positively affect tumor progression. Accordingly, FAP-1 has been reported to inhibit FAS-mediated apoptosis in pancreatic adenocarcinoma [104,105] and melanoma [106]. Interestingly, human melanoma cells silenced for FAP-1 show increased surface FAS expression and respond to recombinant FAS ligand (FasL) treatment by the induction of apoptosis [106]. Contrary to the possibility of blocking FAP-1 for the treatment of melanoma, there is also evidence that FAP-1 can act as a tumor suppressor in some cancer types. For example, reduced PTPN13 mRNA expression due to promoter hypermethylation or allelic loss has been observed in gastric and hepatocellular carcinomas [107,108]. Such a diversity of functions described for FAP-1, with positive and negative roles in a context-dependent manner, could be explained considering that it is among the largest intracellular PTPs, containing eight domains [109]. Despite the demonstrated role of this PTP in melanoma, to date, at least to our knowledge, no therapeutic approaches have been developed to inhibit FAP-1 in this tumor type, leaving open the possibility of designing new inhibitors.

### 2.4. Mitogen-Activated Protein Kinase Phosphatase-1 (MKP1)

**Figure 2:**
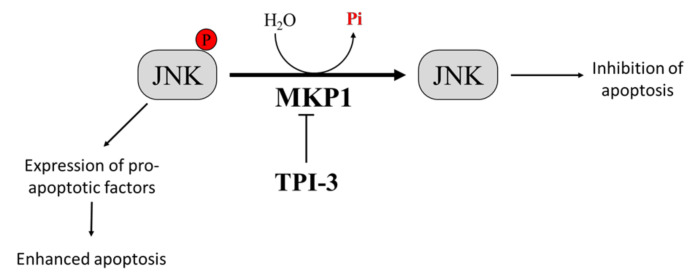
Effect of Mitogen-Activated Protein Kinase Phosphatase-1 (MKP1) targeting on melanoma cells. Overexpression of MKP1 phosphatase in melanoma cells contributes to enhance resistance toward anticancer drugs. For this reason, MKP1 inhibition is sufficient to enhance cancer cell death in culture and to sensitize cancer cells towards cytotoxic drugs. Among the substrates of MKP1, there is JNK. MKP1 dephosphorylates JNK, inhibiting its activation, and thereby avoiding apoptosis.

MKP1 is a member of the threonine-tyrosine dual-specificity phosphatase family. MKP1 targets different members of the MAPK family that regulate cell proliferation and apoptosis, including extracellular signal-regulated kinase (ERK), c-Jun N-terminal kinase (JNK), and p38 MAPK [110]. Different evidence demonstrates that MKP1-mediated JNK dephosphorylation/inactivation is essential to protect tumor cells from anticancer drug-induced apoptosis [111,112]. Interestingly, melanoma patient survival analyzed by the Kaplan–Meier curve describes a correlation between high levels of MKP1 and poor prognosis [71,113]. Therefore, the inhibition of MKP1 may be an effective strategy to enhance the activity of antitumor therapy [114,115]. Promising results in this context come from a study by Kundu and co-workers, who demonstrated that by combining the interferon-α2b (IFN-α2b) with a new selective MKP1 inhibitor, tyrosine phosphatase inhibitor-3 (TPI-3), it is possible to obtain better results than those achieved with IFN-α2b or TPI-3 alone in inhibiting melanoma growth both in vitro and in a xenograft nude mice model. Interestingly, the authors reported that TPI-3 is a well-tolerated compound and that mice treated with TPI-3 alone did not experience loss of weight, abnormalities in behaviors, or anatomic alterations [116] (Figure 2). All together, these findings suggest that therapeutic strategies based on the treatment with MKP1 inhibitors could contribute to improve the prognosis of patients affected by tumors expressing high MKP1 levels, such as melanoma.

### 2.5. Phosphatase of Regenerating Liver (PRL)

The family of PRL consists of three members, namely PRL1, PRL2, and PRL3, and is a unique class of oncogenic dual-specificity phosphatases (alternatively known as protein tyrosine phosphatase 4A, PTP4A). Kaplan–Meier curve analysis of patients affected with melanoma shows a clear correlation between PRL phosphatase expression and poor survival [71,117,118].

Despite their role in cancer being well documented, the molecular functions of these proteins are still not completely understood [119]. PRL3 modulates different signaling pathways involving p53, MAPK, protein kinase B (PKB, also known as AKT), mammalian target of rapamycin (mTOR), signal transducer and activator of transcription 3 (STAT3), FAK, and vascular endothelial growth factor (VEGF), hence positively acting on tumor cell proliferation and aggressiveness [120]. PRL3 is also able to dephosphorylate the PI (4,5) P2 phosphoinositide, thereby contributing to modulation of the tumoral phenotype [121].

In addition, PRL3 promotes cell motility, invasion, and metastasis formation through different mechanisms, including its mutual activation involving the kinase Src [122], the accumulation of MMO14 matrix metalloprotease [123], and the downregulation of the tumor suppressor phosphatase and tensin homolog (PTEN), with consequent epithelial–mesenchymal transition [124]. Interestingly, in a very recent paper, PRL2 overexpression was also correlated with PTEN downregulation and poor patient survival. In particular, the authors proved that PRL2 directly downregulates PTEN by dephosphorylating its Tyr336 residue, thus increasing PTEN ubiquitination and degradation [125].

Overexpression of PRL3 has been demonstrated in many different solid tumors, including metastatic melanoma [30]. This finding was further confirmed by Wu and colleagues, who demonstrated a higher expression of PRL3 in the metastatic melanoma cell line B16-BL6 with respect to its less metastatic counterpart B16 cells, highlighting a clear role of PRL3 in promoting metastasis formation [126]. Daouti and co-authors established that while ectopic PRL3 overexpression induces cell transformation and increases motility and invasiveness, PRL3 silencing prevents anchorage-independent cell growth in soft agar [30]. The authors suggested that the adaptor protein p130Crk-associated substrate (p130Cas) is involved in this mechanism. Specifically, they demonstrated that treatment with thienopyridone, a selective inhibitor of all the three PRL isoforms, induces p130Cas and FAK cleavage, leading to the induction of caspase-mediated cell apoptosis and cancer cell *anoikis* [30].

Coherently, it was also shown that siRNA-mediated PRL3 depletion is able to inhibit the metastatic potential of B16-BL6 mouse melanoma cells both in vitro and in vivo [127].

Even if it has been known for a long time that a correlation exists between high PRL3 expression and metastatic risk in patients with uveal melanoma [128], only recently has a specific role for PRLs been recognized in this aggressive and metastatic tumor. In particular, collapsin response mediator protein 2 (CRMP2), a protein affecting microtubule dynamics, protein endocytosis, and vesicle recycling, has been described as a new target for PRL3 phosphatase activity. Specifically, PRL3 dephosphorylates CRMP2 on Thr514, thus enhancing cell invasiveness [129].

**Figure 3:**
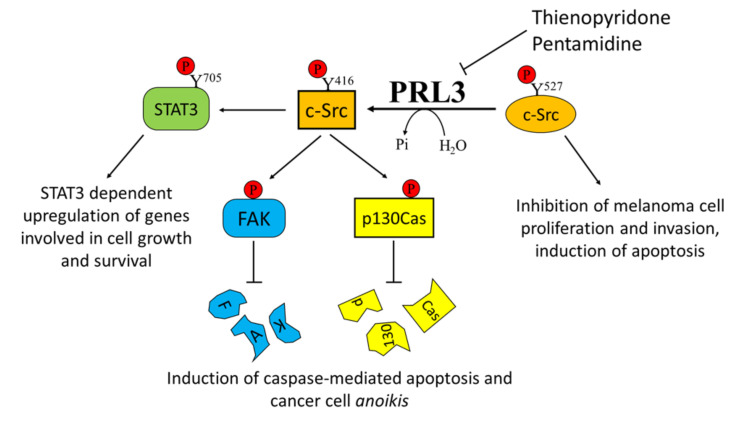
Effects of Phosphatase of Regenerating Liver-3 (PRL-3) targeting on melanoma progression. Elevated PRL-3 leads to Src activation through the downregulation of the synthesis of C-terminal Src kinase protein, which in turn leads to tyrosine phosphorylation of several proteins, including STAT3, FAK, and p130Cas. Thienopyridone and pentamidine derivatives, which act as PRL3 inhibitors, are effective in inhibiting melanoma cell proliferation, survival, and migration.

Considering the key role of PRL3 in mediating melanoma cell motility and metastasis formation, several attempts have been performed in order to select specific PRL3 inhibitors [119]. Pathak and colleagues identified pentamidine [1,5-di(4-amidinophenoxy)pentane] as a relatively specific inhibitor of PRLs and tested its activity on several cancer cell lines, including the WM9 melanoma-derived cell line. Interestingly, pentamidine was also tested in nude mice, where it was able to induce marked tumor cell necrosis in engrafted WM9 human melanoma cells, without any obvious side effects [130]. In addition, the previously mentioned thienopyridone is another promising inhibitor of PRLs that has been demonstrated to be effective in reducing the aggressiveness of melanoma cells by affecting their metastatic potential [30] (Figure 3).

A possible alternative approach is based on the targeting of PRL1 trimer formation, a mechanism necessary for PRL1-mediated cell proliferation and migration [131]. Using a computer-based virtual screening, different specific compounds were selected as PRL1 trimerization inhibitors. Interestingly, one of these compounds, referred to as “Cmpd-43”, displayed a strong anticancer activity both in vitro and in vivo in a murine xenograft model of melanoma [132].

Even if further efforts are needed to improve both the effectiveness of the inhibitors described and to reduce their side effects, the reported results suggest that PRLs could be an optimal target to reduce melanoma aggressiveness.

An event that should be considered when developing new treatments targeting PRLs is its interaction with the CNNM complexes (cyclin-M family, also termed cyclin and cystathionine β-synthase (CBS) domain magnesium transport mediators). This interaction is a key node in the regulation of magnesium homeostasis [133], and cells that overexpress PRLs in complex with CNNMs accumulate intracellular magnesium [134], which favors tumor proliferation and migration [135].

A possible more promising approach has very recently been developed using a humanized antibody (PRL3-zumab) that is able to target externalized PRL3 protein on different human liver and gastric tumor cell lines, used in an orthotopic tumor model in nude mice [136]. This antibody is currently under investigation in a phase 1 clinical trial on a wide range of solid tumors and hematological malignancies (Trial Number: NCT03191682; Table 1).

### 2.6. Src Homology Region 2 Domain-Containing Phosphatase-2 (SHP-2)

SHP-2, also termed tyrosine-protein phosphatase non-receptor type 11, is encoded by the PTPN11 gene [137]. SHP-2 contains two tandem SH2 domains, which act as phospho-tyrosine-binding domains and mediate the interaction of the tyrosine phosphatase with its substrates [137]. SHP-2 is auto-inhibited in the resting state, since the N-terminal SH2 domain binds to the catalytic cleft of the PTP domain, thereby blocking the access of SHP-2 substrates to the active site. Upon binding to target phospho-tyrosine residues, the N-terminal SH2 domain is released from the PTP domain and thus the enzyme is catalytically activated by reverting its auto-inhibited conformation [138].

SHP-2 is ubiquitously expressed and plays a key role in different cell signaling events, such as mitogenic activation, metabolic control, transcriptional regulation, and cell migration [139].

SHP-2 is the first tyrosine phosphatase identified as an oncogene in juvenile myelomonocytic leukemia, myelodysplastic syndromes, and acute myeloid leukemia [140]. It is remarkable that the gain-of-function mutations in SHP-2, leading to hyper-activated/deregulated mutants of the enzyme, occur in about 50% of Noonan syndrome patients [141]. Importantly, increased SHP-2 expression is a prognostic and a predictive marker of several malignancies and plays a key role in melanoma [142,143,144,145,146,147,148,149]. Indeed, this PTP has been found to be overexpressed and mutated in samples derived from melanoma patients, correlating with a strong metastatic phenotype and a poorer prognosis [33,34,35]. These findings were further confirmed by the Kaplan–Meier curve analysis, which revealed a strong correlation between higher expression levels of SHP-2 and poor overall survival in melanoma patients [71,150].

Due to the involvement of SHP-2 in multiple growth factor-mediated oncogenic pathways, such as the Ras/ERK1/2 pathway, and to its fundamental role in several tumors, inhibition of SHP-2 is considered to have broad therapeutic applications in the treatment of various cancers, including melanoma [151].

The PTP inhibitor sodium stibogluconate (SSG), a drug used in the treatment of leishmaniasis [152] and identified as an inhibitor of both SHP-1 and SHP-2 [153], increases interferon-α (IFN-α)-induced signal transducer and activator of transcription 1 (STAT1) tyrosine phosphorylation and has been shown to synergize with IFN-α to inhibit WM9 human melanoma tumor growth in nude mice [154]. Accordingly, Win-Piazza and co-workers demonstrated that suppression of SHP-2 increases the antitumor activity of IFN-α2b in A375 melanoma tumor xenografts. Indeed, IFN-α2b exerts antiproliferative effects on A375 cells through STAT1/STAT2 tyrosine phosphorylation, which is negatively regulated by SHP-2. In keeping with these data, treatment with the SHP-2 inhibitor, SPI-112, increases the IFN-α2b-stimulated STAT1 phosphorylation and inhibits A375 cell growth [155].

Furthermore, Soong and colleagues revealed a peculiar role of SHP-2 in melanocytes. Specifically, Plexin B1 and tyrosine protein kinase Met (MET) assemble in an oligomeric receptor-receptor complex in melanocytes and Semaphorin-4D (Sema4D) increases this association. The consequent MET activation correlates with the transformation of melanocytes to melanoma [156]. SHP-2 mediates, at least in part, the effects downstream to the MET receptor, and this phosphatase is required for the activation of the MAPK and AKT signaling pathways in response to hepatocyte growth factor (HGF) [157,158]. The blockade of SHP-2 phosphatase activity with the inhibitor NSC-87877 reduces HGF-induced MET activation and subsequently ERK1/ERK2 and AKT phosphorylation, suggesting an important role for SHP-2 in transducing proliferative and prosurvival signals in melanocytes [156]. Consequently, inhibition of SHP-2 can be proposed as a novel target to halt the transformation of melanocytes in melanoma.

Furthermore, SHP-2 acts as an oncogene in BRAF wild-type (either NRAS mutant or wild-type) melanoma cells. Indeed, both silencing of the activated SHP-2 E76K mutant or the administration of the allosteric SHP-2 inhibitor, SHP099, causes regression of the established melanoma, thereby suggesting that SHP-2 could be considered as a therapeutic target for BRAF wild-type melanoma [34].

It is widely described that HGF confers resistance to the BRAF inhibitor Vemurafenib in BRAF-mutant melanoma cells [159]. Interestingly, recent evidence underlines that SHP-2 is necessary to mediate this mechanism of resistance. Indeed, Prahallad and co-workers revealed that SHP-2 knockout clones of SK-Mel888 BRAF(V600E) mutant melanoma cells were unable to confer Vemurafenib resistance, following HGF, fibroblast growth factor 9 (FGF9), and stem cell factor (SCF) exposure [56]. Furthermore, it has been demonstrated that SHP-2 also drives adaptive resistance to RAS viral (v-raf) oncogene homolog (RAF) and MEK inhibitors in other tumor types [160]. Accordingly, Ahmed and co-authors proved that co-targeting of MEK and SHP-2 could serve as a powerful therapeutic approach in triple-negative breast cancer and showed that SHP-2 inhibition impairs adaptive resistance to Vemurafenib in a subset of BRAF(V600E) colorectal and thyroid cancers. These results suggest that SHP-2 blockade successfully overcomes adaptive resistance to BRAF and MEK inhibitors in a defined subgroup of ERK-dependent tumors, keeping the possibility open for exploiting this strategy for melanoma treatment.

**Figure 4:**
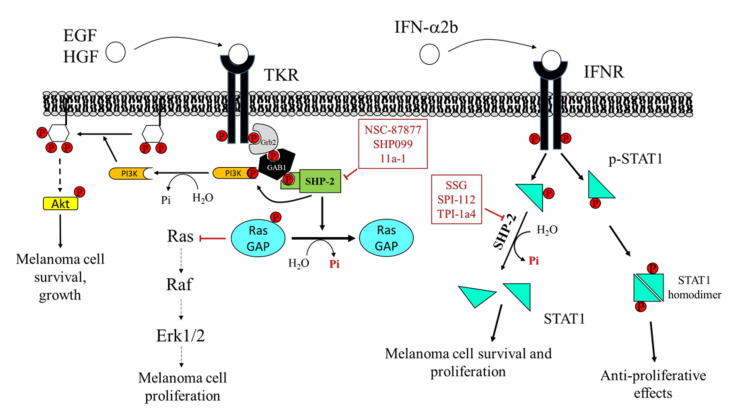
Effects of Src Homology Region 2 Domain-Containing Phosphatase-2 (SHP-2) targeting on melanoma cells. Left: SHP-2 has a key role in promoting the proliferation and survival of melanoma cells. SHP-2 dephosphorylates RasGAP, an inhibitor of Ras. Therefore, SHP-2, by activating Ras, promotes the RAS/RAF/ERK1/2 pathway, which sustains melanoma cell proliferation. Right: SHP-2 dephosphorylates GRB2-associated-binding protein 1 (GAB1), which releases PI3K, promoting activation of the PI3K/AKT pathway, melanoma cell growth, and survival. NSC-87877 and SHP099 inhibit SHP-2, impairing melanoma cell proliferation. SHP-2 also has an important role in regulating signaling activated by IFN-α2b. SHP-2 dephosphorylates STAT1, hindering its dimerization and its migration into the nucleus, where it stimulates the transcription of several genes, resulting in melanoma cell growth arrest. Overexpression of SHP-2 in melanoma cells blunts the response to IFN-α, favoring melanoma cell survival and dissemination. SSG, SPI-112, and TPI-1a4 are potent inhibitors of SHP-2, enhancing the anti-proliferative activity of IFN-α.

Moreover, SHP-2 acts as a scaffold protein recruiting growth factor receptor-bound protein 2/Son of Sevenless (GRB2/SOS) complex to the membrane and promoting RTK-mediated RAS activation [161,162]. It is noteworthy that the allosteric SHP-2 inhibitor SHP099 stabilizes the phosphatase in its inactive conformation [22], thus preventing the assembly of SHP-2 with other adaptor proteins to achieve the complete activation of RTK signaling. In keeping with this, Zhang and colleagues demonstrated that SHP-2 overexpression enhances melanoma MeWo cell viability, motility, and anchorage-independent growth, through positive regulation of the ERK1/2 and PI3K/AKT pathway [35]. Accordingly, SHP-2 knockdown is able to revert these effects. Indeed, the specific SHP-2 inhibitor 11a-1 [163], an indole salicylic acid inhibitor, reduces the aforementioned phenomena in melanoma cells by downregulating the SHP-2-mediated ERK1/2 and AKT signaling pathways. Moreover, in vivo experiments demonstrated that 11a-1 significantly reduces xenografted melanoma tumor growth (Figure 4).

Overall, following the clear correlation between high expression levels of SHP2 and poorer survival of melanoma patients, several findings strongly suggest that SHP-2 may act as a targetable substrate against melanoma. In this perspective, SHP-2 inhibitors can be proposed as novel therapeutic approaches for melanoma treatment [35].

In keeping, TNO155, a recently discovered orally bioavailable SHP-2 inhibitor with antitumor activity in xenograft models [164], is currently in clinical trials for the treatment of solid tumors, including melanoma (Trial Number: NCT03114319; Table 1).

## 3. Role of PTPs in Immune Melanoma Cell Infiltrate

Melanoma is reported as one of the most immunogenic tumors characterized by a crosstalk between cancer cells and immune cells, which strongly affects cancer progression and metastasis [165]. Indeed, during melanomagenesis, activated T cells are recruited to the melanoma microenvironment through a “homing” mechanism, in order to recognize melanoma antigens and attack tumor cells, finally inducing cell death by the apoptosis pathway or the granule exocytosis pathway [166,167]. Interestingly, patients affected by melanoma with high CD8+ T cell infiltrate both in primary tumor and metastasis have better outcomes [168]. Targeting of the host immune response in melanoma with immunotherapies is one of the most interesting approaches used in the last decades to fight this malignancy [169]. Metastatic melanoma is mainly treated with Food and Drug Administration (FDA)-approved immune checkpoint inhibitors, such as Ipilimumab, Pembrolizumab, and Nivolumab [170,171,172,173]. However, many patients fail to respond to immunotherapy and frequently develop primary or secondary resistance, highlighting the need for supportive therapies to fight melanoma [174,175]. Several studies underlined that PTPs can exert an important role in modulating the immune infiltrate, thereby affecting melanoma growth. Hence, inhibition of these PTPs might represent a novel strategy to be combined with the already consolidated immunotherapies.

### 3.1. Src Homology Region 2 Domain-Containing Phosphatase-1 (SHP-1)

SHP-1, also known as protein tyrosine phosphatase non-receptor type 6 (PTPN6), encoded by the PTPN6 gene, belongs to the family of non-receptor PTPs. This enzyme localizes to the cytosol and it is primarily expressed in hematopoietic cells, whereas it is present only at low levels in epithelial cells [176,177]. Similarly to SHP-2, SHP-1 is characterized by two tandem N-terminal SH2 domains that regulate the enzyme activity. The three-dimensional crystal structure of ligand-free SHP-1 revealed that this tyrosine phosphatase displays an auto-inhibited conformation [178].

SHP-1 was initially classified as a tumor suppressor phosphatase [179] since its silencing, due to hypermethylation of CpG islands in the PTPN6 promoter region, is frequently associated with a poor prognosis and worse outcome in different types of cancers [180,181,182,183,184,185,186,187,188,189]. Indeed, several studies underlined that this enzyme controls cell cycle progression by impairing PDGF and insulin receptor signaling [190,191]. Moreover, Nagakami and co-authors demonstrated that in endothelial cells, SHP-1 activation by tumor necrosis factor-alpha (TNF-α) inhibits VEGF- and epidermal growth factor (EGF)-mediated proliferation [192].

Despite the widely described tumor suppressor activity of SHP-1, recent studies demonstrated that this phosphatase could also act as an oncogene [193,194]. In particular, growing evidence highlights that SHP-1 is involved in modulation of the tumor microenvironment and impacts on immune infiltrate activation.

**Figure 5:**
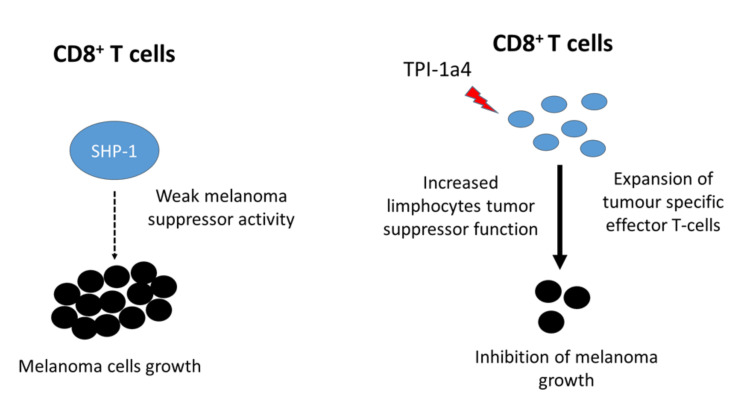
Effects of Src Homology Region 2 Domain-Containing Phosphatase-1 (SHP-1) targeting in T cells. Tumor-infiltrating lymphocytes express SHP-1, which regulates the TCR-driven T cell activation threshold and inhibits early events after TCR triggering. The SHP-1 inhibition increases interaction of CD8^+^ T-cells with antigen presenting cells (APCs), leading to reduced activation thresholds and increased proliferation of antitumor T cells. Therefore, the inhibition of SHP-1 improves the ability of immune cells to suppress melanoma cell growth.

Specifically, SHP-1 is a key negative regulator of cytokine signaling and immune cell activity [195,196]. This tyrosine phosphatase limits T cell responsiveness by directly targeting T cell receptor (TCR) chain-ζ or downstream effectors, including the lymphocyte-specific protein tyrosine kinase (Lck), the zeta-chain-associated protein kinase 70 (ZAP70), the proto-oncogene vav (Vav), and PI3K [176,197]. Recently, Watson and co-authors highlighted that SHP-1 deficiency increases the ability of adoptively transferred CD8^+^T cells to impair tumor growth [198]. In addition, an enhanced in vivo cytotoxicity of naive SHP-1-deficient T cells was observed, highlighting the validity of targeting SHP-1 expression to boost human CD8^+^T cell functionality [199,200] (Figure 5).

Considering the relevance of the immune infiltrate in supporting melanoma progression, targeting SHP-1 with specific inhibitors could be a promising novel strategy to improve the efficacy of cytokine therapy and immunotherapy, which are among the most successful approaches in clinical use for melanoma treatment [201,202]. For this purpose, different SHP-1 inhibitors have been developed, displaying antitumor potential in advanced cancer patients, including melanoma patients [153,203,204,205], as demonstrated by two different clinical trials (Trial Number: NCT00629200 and NCT00498979; Table 1). In this context, Yi and colleagues demonstrated that SSG inhibitor synergizes with IFN-α to overcome IFN-α resistance in different human cancer cell lines and extinguishes IFN-α-refractory WM9 human melanoma tumors in nude mice without displaying adverse effects [154].

More recently, a novel SHP-1 tyrosine phosphatase inhibitor-1 (TPI-1) was identified through the screening of a library of 34,000 drug-like compounds. TPI-1 was more effective than SSG in SHP-1 inhibition, immune cell activation, and antitumor potential [205]. Furthermore, Kundu and co-workers revealed that TPI-1 increases the expression of interferon-γ (IFN-γ), a cytokine produced during the activation of antitumor cells, in vitro, in mouse splenocytes, and in human peripheral blood. Interestingly, these authors also found that TPI-1 has anticancer potential on B16 melanoma thanks to the described induction of IFN-γ and demonstrated that B16 tumor growth is inhibited following TPI-1 treatment. Moreover, the antitumor activity of TPI-1a4, a TPI active analogue, was evaluated on the UV-induced K1735 murine melanoma with promising results [205].

Furthermore, Ramachandran and co-workers described the central role of SHP-1 in dendritic cell (DC) function. Indeed, this phosphatase inhibits numerous downstream effectors of multiple receptors, such as the nuclear factor kappa-light-chain-enhancer of activated B cells (NFkB), the activator protein 1 (AP-1), ERK, and JNK, as well as cytokine production in DCs. Accordingly, SSG-mediated inhibition of SHP-1 promotes proinflammatory cytokine production and increases the survival and migration of dendritic cells. Since DC signaling is required for the initiation of T cell immunity, SHP-1 is also able to reduce the ability of DCs to induce antigen-specific T cell proliferation. Finally, these authors assessed that SHP-1 inhibition in DCs could enhance their potency as antitumor vaccines. It is notable that mice bearing B16F10 melanoma, vaccinated with SHP-1-silenced DCs or with SHP-1 dominant negative DCs, showed a significantly slower tumor growth if compared to controls. Interestingly, this result suggests that inhibition of SHP-1 in DCs can improve their efficacy as in vivo antitumor vaccines against melanoma [206].

Moreover, a recent study demonstrated that SHP-1 inhibition in combination with immune checkpoint blockade treatment (anti-PD1 and anti-CTLA4) increases the recruitment and the effectors’ function of low-affinity T cells, finally leading to melanoma tumor regression by increasing the frequency of IFN-γ-producing endogenous antitumor CD8^+^T cells [207]. Collectively, this evidence highlights the role of SHP-1 as an oncogene in the tumor microenvironment, specifically in the immune cell population, suggesting that it may be a promising target to enlarge the repertoire of T cells sensitive to checkpoint blockade, finally leading to enhanced control of melanoma growth.

Notably, new efforts are needed to modulate SHP-1 activity in the selected immune cell population, which would be a significantly better strategy to impair tumor growth, rather than globally inhibit SHP-1 activity, independently of the cell subset. This approach could be particularly suitable in melanoma treatment, where immunotherapy is becoming increasingly important [208].

### 3.2. Src Homology Region 2 Domain-Containing Phosphatase-2 (SHP-2)

**Figure 6:**
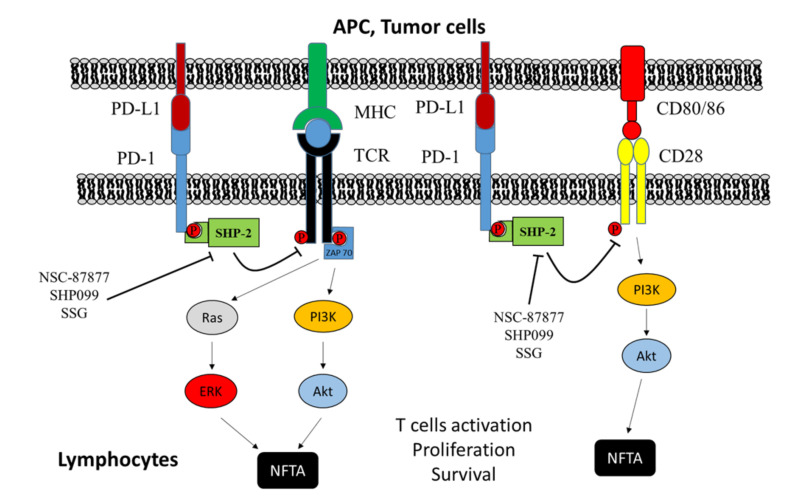
Targeting of SHP-2 in immune cells. After binding with PD-L1 ligand exposed by cancer cells or APC cells, PD-1 undergoes activation and recruits SHP-2, which can dephosphorylate ZAP70 or activate CD28 immune receptor. By this mechanism, SHP-2 inhibits signaling originated by TCR and CD28, thereby leading to a reduction in TCR-mediated interleukin-2 (IL-2) production and the impairment of T cell proliferation. Conversely, the treatment with SHP-2 inhibitors, such as NSC-87877, SHP099, and SSG, enhances lymphocyte activation and cytokine release, and stimulates lymphocyte proliferation, inducing melanoma cell death.

Recent evidence suggests a role of SHP-2 in tumor immunity. Specifically, following engagement with its ligands, mainly PD-L1, programmed cell death protein 1 (PD-1) is activated and recruits SHP-2 in the proximity of TCR and CD28. Consequently, SHP-2 dephosphorylates and decreases TCR and CD28 pathways, leading to the inhibition of T cell proliferation and activation, ultimately causing activated T cell death [209]. In keeping with this, Wu and co-workers recently showed that B16 melanoma cell-derived exosomes deliver SHP-2 to tumor-infiltrating lymphocytes, thus suppressing their function and inhibiting their proliferation [210]. Moreover, it has been demonstrated that SHP-2 suppression in macrophages may promote a Th1-dominant tumor immune microenvironment, which is advantageous to suppress melanoma growth. Indeed, the deletion of SHP-2 increases macrophage production of chemokine (C-X-C motif) ligand 9 (CXCL9) in response to IFN-γ and tumor cell-derived cytokines, promoting T cell infiltration into the tumor [211]. Other evidence highlighting the importance of SHP-2 targeting in tumor-based immunotherapy comes from the studies of Ramesh and co-workers [212]. These authors demonstrated that the treatment with dual-inhibitor-loaded nanoparticles (DNTs), which simultaneously inhibit macrophage colony-stimulating factor 1 receptor (CSF1R) and SHP-2 pathways, results in an efficient re-polarization of M2 macrophages to their active M1 phenotype. Moreover, DNTs display antitumor activity without any toxicity in melanoma in both in vitro and in vivo settings [212]. In conflict with previous evidence, Zhang and co-authors highlighted that the expression of SHP-2 in CD4^+^ T cells exerts tumor-suppressing effects on melanoma. Indeed, they demonstrated that SHP-2 deletion in CD4^+^ T cells potentiates melanoma progression and promotes metastasis in mice [213] (Figure 6).

Even if the role of SHP-2 in modulating antitumor activity of immune infiltrate needs to be better investigated, reported results suggest that targeting SHP-2 in immune cells may be a promising approach for melanoma treatment.

### 3.3. Tyrosine-Protein Phosphatase Non-Receptor Type 2 (PTPN2)

PTPN2, also known as T-cell protein tyrosine phosphatase (TC-PTP), is ubiquitously expressed and has important functions [214] in modulating immune cell signaling [215], inflammatory responses [216], hematopoietic stem cell renewal [217], insulin signaling [218], and leptin regulation [219]. Importantly, several recent studies outlined a key role of PTPN2 in modulating oncogenic signaling. While few papers attribute a tumor-suppressive function to PTPN2 [220,221,222,223], many studies support its oncogenic role. For example, PTPN2 has been found to be overexpressed in B cell lymphomas, where its upregulation is under the control of MYC [224]. In addition, PTPN2 promotes IGF-2 induced migration in MCF-7 breast cancer cells [225].

Using Clustered Regularly Interspaced Short Palindromic Repeats/CRISPR associated protein 9 (CRISPR/Cas9) genome editing, Manguso and co-workers recently performed a pooled loss-of-function genetic screening in mice tumors derived from transplantable B16 melanoma cells. Mice were treated with immunotherapy in order to discover new potential targets involved in the resistance to this approach. Interestingly, among the genes depleted in immunotherapy-treated mice, PTPN2 was one of the most representative. Specifically, loss-of-function of PTPN2 sensitized B16 cells to immunotherapy in vivo and increased the sensitivity to T cell immunity in a *Braf/Pten* melanoma model, without affecting cell viability and growth in the absence of T cells. It is noteworthy that the re-expression of PTPN2 in PTPN2-null tumors abrogated the response to immunotherapy in vivo and the overexpression of PTPN2 in B16 cells promoted immunotherapy resistance. Moreover, the composition of the immune cell population in the tumor microenvironment was strongly altered in PTPN2-null tumors displaying a higher number of activated and cytotoxic CD8^+^T cells and γδ^+^ T cells. In addition, loss of PTPN2 increased antigen presentation and sensitivity to CD8^+^T cells. In order to dissect the mechanism by which PTPN2 acts as an oncogene in melanoma and how its loss correlates with increased sensitivity of tumor cells to immunotherapy, it is important to underline that the tyrosine phosphatase inhibits IFNγ signaling by dephosphorylating STAT1 and JAK1. Indeed, PTPN2 deletion in B16 tumor cells enhanced IFNγ signaling, caused a strong change in the expression profile of IFNγ response gene, and increased phosphorylation of STAT1, thereby reducing tumor growth of PTPN2-null mice [226].

Moreover, a very recent study disclosed a new role of PTPN2 as a key regulator of the differentiation of the terminally exhausted CD8^+^T cell subpopulation by attenuating type I interferon signaling. Indeed, PTPN2 loss in the immune system subpopulation, CD8^+^T cells, induces PD-1 checkpoint blockade response to B16 tumors [227].

Despite no PTPN2 inhibitors having been extensively studied to date, the previously reported promising results revealing the oncogenic role of PTPN2 strongly suggest the necessity to design selective inhibitors of this phosphatase in order to potentiate the sensitivity of melanoma to the effects of immunotherapy.

## 4. Discussion

The discovery that mutated or constitutively activated tyrosine kinases contribute to melanocyte transformation pointed the attention to the importance of altered tyrosine phosphorylation in supporting different aspects of melanoma progression. This evidence paved the way for the development of tyrosine kinase inhibitors for the treatment of the unresectable form of melanoma [228]. Hence, many tyrosine kinase inhibitors have been synthesized in the past decades, and quickly approved for the treatment of patients affected by advanced metastatic melanoma. Although this therapeutic approach has produced clear benefits [229], clinical data revealed that a significant percentage of patients expressing mutated forms of oncogenic tyrosine kinases develop resistance to the treatment over the time, highlighting the need to find alternative therapeutic approaches [230].

A possible solution to this issue has recently emerged from the modulation of the activity of PTPs. Indeed, although many members of the PTP family behave as a tumor suppressor, several studies underscored a key role for PTPs as tumor promoters in different types of cancer [8]. This scenario suggested that the targeting of PTPs could be a promising alternative strategy to fight cancer progression. Despite the difficulties arising from the fact that all PTPs share the same charged active site, many advances have been made in the development of specific, cell-permeable, and bioavailable PTP inhibitors, mainly allosteric ones [231]. The first study about the possibility of inhibiting PTPs for melanoma treatment came from the late 1990s when Steinman and collaborators reported that the administration of different PTPs inhibitors, such as sodium orthovanadate and phenylarsine oxide, strongly inhibited melanoma metastasis formation, thus reducing tumor aggressiveness [232]. Preclinical studies subsequently revealed that some molecules targeting PTPs are able to slow down proliferation and reduce the metastatic dissemination of many aggressive forms of cancers without producing side effects, suggesting the possibility of introducing these compounds in the therapy against melanoma [21,231].

The evidence reported in this review confirms this hypothesis and shows that some of the PTPs previously described to have oncogenic functions also play an important role in promoting melanoma cell proliferation and survival [30,31,32,33,34,35]. In addition, some of these PTPs are implicated in the inactivation of the immune response, contributing to immune surveillance evasion of melanoma cells [195,196,209,226,227]. The relevance of PTPs in sustaining melanoma aggressiveness is further confirmed by tests carried out on mice xenografted with melanoma cells, revealing that treatment with PTPs inhibitors impairs proliferation, migration, and invasiveness of cancer cells [116,130], and enhances the antitumoral immune response [154,205,206,226]. Interestingly, many of the PTP inhibitors proposed above have been designed to overcome the highly conserved and positively charged nature of PTP active sites, thus ensuring a good specificity for their targets. For example, the SHP-2 inhibitor 11a-1, demonstrated to be effective against melanoma both in vitro and in vivo [163], has an IC50 over 5-fold more selective for SHP2 than any other PTPs tested. This specificity is correlated to its bidentate structure, which facilitates the access and the anchorage to both the SHP2 active site and a unique peripheral binding site, increasing its selectivity and potency [163]. The step-by-step modification strategy is also successful for the identification of new inhibitors. This approach, by optimizing the previously proposed CDC25B inhibitor NSC28620, led Cerchia and co-workers to the identification of the main structural requirements necessary to obtain the optimal inhibitory activity and specificity to design selective inhibitors against CDC25B [83].

Moreover, it is important to notice that some PTPs are simultaneously overexpressed in both cancer and immune cells, often displaying opposite functions. In these cases, it would be necessary to have some caution before proposing them as a possible therapy, evaluating the possibility of targeting the enzymes exclusively in one of the two populations.

For instance, it has been reported that LMW-PTP has a positive role in regulating the activation of lymphocytes [233,234], while it also promotes resistance of melanoma cells toward cytotoxic drugs [31]. As a consequence, the treatment of patients affected by melanoma with LMW-PTP inhibitors could induce a paradoxical effect, inhibiting on the one side, the proliferation of cancer cells, but on the other, impairing the immune response.

Different is the case of SHP-2, which plays a key role in sustaining melanoma cell survival, and behaves, at the same time, as a negative regulator of T lymphocytes [235]. Therefore, in this case, treatment with SHP-2 inhibitors could generate a synergistic effect, dampening melanoma cell growth [22] and at the same time potentiating the activation of immune cells [236].

In conclusion, in our opinion, future studies should be focused, on the one hand, on clarifying the physiological roles of PTPs in order to predict the efficacy of new targeted therapies based on their inhibition and, on the other, to synthesize more specific PTP inhibitors with a view to generating novel potential antitumoral drugs.

## 5. Conclusions

In synthesis, information collected in this review demonstrates that PTPs represent interesting molecules that can be targeted to fight the most aggressive forms of melanoma. PTP inhibitors could be successfully used, alone or in combination, to arrest melanoma progression while also potentiating antitumor immune surveillance. Based on the promising results obtained so far, PTP inhibitors could be proposed as a potential adjuvant therapy for the treatment of melanoma.

## Figures and Tables

**Table 1:** Protein tyrosine phosphatase (PTP) inhibitors involved in clinical trials for melanoma treatment.

Trial Number	Compound	Target	Disease	Status
NCT03191682	PRL3-ZUMAB	PRL3	Solid Tumors and Hematologic Malignancies	Phase I
NCT03114319	TNO155	SHP-2	Non-Small Cell Lung Cancer; Esophageal Squamous Cell Cancer (SCC); Head/Neck SCC; Melanoma	Phase I
NCT00629200	Sodium stibogluconate	SHP-1	Malignant melanoma	Phase I completed
NCT00498979	Sodium stibogluconate	SHP-1	Malignant melanoma	Phase I completed

PRL3: Phosphatase of Regenerating Liver-3; SHP-2: Src Homology Region 2 Domain-Containing Phosphatase-2; SHP-1: Src Homology Region 2 Domain-Containing Phosphatase-1.
